# Identification of cuproptosis-related molecular subtypes and a novel predictive model of COVID-19 based on machine learning

**DOI:** 10.3389/fimmu.2023.1152223

**Published:** 2023-07-17

**Authors:** Hong Luo, Jisong Yan, Dingyu Zhang, Xia Zhou

**Affiliations:** ^1^ Department of Tuberculosis and Respiratory, Wuhan Jinyintan Hospital, Tongji Medical College of Huazhong University of Science and Technology; Hubei Clinical Research Center for Infectious Diseases; Wuhan Research Center for Communicable Disease Diagnosis and Treatment, Chinese Academy of Medical Sciences; Joint Laboratory of Infectious Diseases and Health, Wuhan Institute of Virology and Wuhan Jinyintan Hospital, Chinese Academy of Sciences, Wuhan, China; ^2^ The First Affiliated Hospital of USTC, Division of Life Sciences and Medicine, University of Science and Technology of China (USTC), Hefei, Anhui, China; ^3^ Center for Translational Medicine, Wuhan Jinyintan Hospital, Tongji Medical College, Huazhong University of Science and Technology (HUST), Wuhan, Hubei, China; ^4^ Department of Critical Care Medicine, Union Hospital, Tongji Medical College, Huazhong University of Science and Technology (HUST), Wuhan, Hubei, China

**Keywords:** COVID-19, SARS-CoV-2, cuproptosis, risk score, immune microenvironment

## Abstract

**Background:**

To explicate the pathogenic mechanisms of cuproptosis, a newly observed copper induced cell death pattern, in Coronavirus disease 2019 (COVID-19).

**Methods:**

Cuproptosis-related subtypes were distinguished in COVID-19 patients and associations between subtypes and immune microenvironment were probed. Three machine algorithms, including LASSO, random forest, and support vector machine, were employed to identify differentially expressed genes between subtypes, which were subsequently used for constructing cuproptosis-related risk score model in the GSE157103 cohort to predict the occurrence of COVID-19. The predictive values of the cuproptosis-related risk score were verified in the GSE163151 cohort, GSE152418 cohort and GSE171110 cohort. A nomogram was created to facilitate the clinical use of this risk score, and its validity was validated through a calibration plot. Finally, the model genes were validated using lung proteomics data from COVID-19 cases and single-cell data.

**Results:**

Patients with COVID-19 had higher significantly cuproptosis level in blood leukocytes compared to patients without COVID-19. Two cuproptosis clusters were identified by unsupervised clustering approach and cuproptosis cluster A characterized by T cell receptor signaling pathway had a better prognosis than cuproptosis cluster B. We constructed a cuproptosis-related risk score, based on PDHA1, PDHB, MTF1 and CDKN2A, and a nomogram was created, which both showed excellent predictive values for COVID-19. And the results of proteomics showed that the expression levels of PDHA1 and PDHB were significantly increased in COVID-19 patient samples.

**Conclusion:**

Our study constructed and validated an cuproptosis-associated risk model and the risk score can be used as a powerful biomarker for predicting the existence of SARS-CoV-2 infection.

## Introduction

Copper, a prevalent metallic element, functions as an indispensable cofactor for bodily enzymes, exerting regulatory influence over numerous physiological processes encompassing energy metabolism, mitochondrial respiration, and antioxidation (1). Cuproptosis, a recently recognized mode of cell death instigated by copper ions, exhibits a distinctive pattern (2). Unlike well-established forms of cell demise such as apoptosis, pyroptosis, necroptosis, and ferroptosis, cuproptosis relies on mitochondrial respiration. Apoptosis, a programmatic process of cellular demise, assumes a significant role in COVID-19. The infection by SARS-CoV-2 activates both intrinsic and extrinsic apoptotic pathways through the viral protein ORF3a. ORF3a triggers the activation of caspase-8 and the cleavage of Bid, thereby instigating the liberation of mitochondrial cytochrome c and the activation of caspase-9 (3). Anomalous expression of apoptosis-related genes and mitochondrial malfunction have been detected in COVID-19 patients, suggesting the involvement of the intrinsic apoptotic pathway (4). COVID-19 is associated with lymphopenia, which manifests as a reduction in CD4+ and CD8+ T-cell subsets. Lymphocyte apoptosis in COVID-19 is contributed to by mitochondrial dysfunction, anomalous mitochondria, and escalated expression of CD95 (5). Necroptosis, a variant of programmatic cellular demise, is induced by SARS-CoV-2 in human lung cells. The virus triggers the phosphorylation of MLKL via RIPK3, consequently culminating in necroptosis. Higher levels of phosphorylated MLKL are discerned on the plasma membrane of infected cells (6). The ripoptosome, comprising caspase-8, FADD, and RIPK1, governs the RIPK3-MLKL-dependent signaling of necroptosis in the absence of caspase-8 activation (7, 8). Pyroptosis, an immensely inflammatory form of cellular demise, is activated in COVID-19 and contributes to the inflammatory response observed in the disease. SARS-CoV can incite the activation of the NLRP3 inflammasome via viral proteins, thereby yielding an ionic imbalance, mitochondrial impairment, production of reactive oxygen species (ROS), and co-activation of NLRP3 (9, 10). The E protein and ORF3a of SARS-CoV activate NLRP3 by triggering the signaling of NF-κB and promoting the ubiquitination of ASC (11, 12). Nonetheless, while the mechanism of NLRP3 activation by SARS-CoV-2 remains incompletely elucidated, the similarities with SARS-CoV hint at a comparable process (13). Ferroptosis, a controlled form of cellular demise characterized by lipid peroxidation, may be implicated in COVID-19. The infection by SARS-CoV-2 incites oxidative stress, inflammation, and perturbation of iron metabolism, culminating in heightened levels of intracellular iron, lipid peroxidation, and depletion of antioxidant systems (14). The targeted modulation of the ferroptosis signaling pathway through inhibitors holds the potential to alleviate the multi-organ damage inflicted by COVID-19. In cuproptosis process, copper directly binds to the lipid-acylated region of the tricarboxylic acid cycle, inducing lipid-acylated protein aggregation and iron-sulfur cluster protein instability, leading to proteotoxic stress, which causes cell death (15). Additionally, several studies have shown copper’s contribution to immunomodulation. Tan et al. found that lysyl oxidase-like 4 (LOXL4) could promote immune evasion in hepatocellular carcinoma cells, which can be eliminated by abolishing LOXL4-mediated PD-L1 presentation by copper chelators (16). Additionally, copper chelating agents greatly boosted the quantity of CD8+ T and natural killer cells that infiltrated tumors (17). Moreover, clioquinol, a copper chelator, effectively reduces the infiltration of CD4 cells, CD8 cells, and CD20 cells, which are immune cells associated with autoimmune encephalomyelitis (18).

Severe Acute Respiratory Syndrome Coronavirus 2 (SARS-CoV-2) is the cause of the coronavirus disease 2019 (COVID-19), which is currently sweeping the world, placing a significant burden on global economic systems and health systems (19). 634 million confirmed cases and 6.6 million fatalities were reported globally as of November 20, 2022 (https://covid19.who.int/). Patients suffering from COVID-19 exhibit immune system abnormalities, such as immune cells and cytokines. It was found that total lymphocytes, CD4+ lymphocytes, and CD8+ lymphocytes were significantly reduced in COVID-19 patients and were more severely impaired in severe cases (20). After treatment, CD4+ and especially CD8+ T lymphocytes were elevated considerably. In addition, CD4+ lymphocytes were more responsive to viral surveillance than CD8+ lymphocytes (21). As for cytokine, it was revealed that IL-2, IL-4, IL-6, and IL-10 were abnormally activated in COVID-19 patients and that IL-6 levels correlated with disease severity (22). Studies have shown a strong link between COVID-19 and several cell death modalities, including apoptosis, pyroptosis, necroptosis, and ferroptosis. However, no relationship between cuproptosis and COVID-19 has been reported (23). Therefore, further research is needed to investigate the function of cuproptosis in COVID-19 and determine how cuproptosis affects the immunological function of lymphocytes in COVID-19.

In this study, we methodically characterized the immunological landscapes in patients with and without COVID-19 and presented the connection between lymphocytes and cuproptosis in these two groups. Then, we identified two distinct cuproptosis subtypes in COVID-19 patients based on the expression levels of 20 cuproptosis-related genes (CRGs). Interestingly, the two subtypes differed in the immune pathway activities and immune cell compositions. We developed a scoring system called the cuproptosis-related risk score (CRRS) based on four CRGs to more accurately measure the cuproptosis level in each patient group. This technique was subsequently examined using two separate clinical manifestation groups and cuproptosis subtypes. Finally, we developed a nomogram based on CRRS and clinical parameters to accurately identify patients with SARS-CoV-2 infection.

## Materials and methods

### Transcriptome data collection and pre-processing

Twelve CRGs were obtained from previously published literature (15). The GSE157103 dataset contains gene expression profiles of 100 patients with COVID-19 and 26 patients without COVID-19 (24). Additionally, this dataset included clinical characteristics such as age, gender, APACHE II and Charlson scores, hospital-free days during a 45-day follow-up (HFD45), ferritin, CRP, D-dimer, procalcitonin, lactate, fibrinogen and, SOFA score. The HFD45 metric assigns a score of zero (0-free days) to patients who have been hospitalized for over 45 days or have passed away during their hospital stay. Conversely, patients with shorter durations of hospitalization and milder disease conditions are assigned higher HFD45 values (25). The GSE163151 dataset, GSE152418 dataset, and GSE171110 dataset are all COVID-19 datasets used to validate CRGs expression patterns and assess the predictive efficiency of CRRS (26–28). The platform’s annotation files were downloaded, the probes were converted into gene symbols, and the expression level of the genes was calculated using the maximum expression level of the duplicate gene symbols.

### Identification of cuproptosis subtypes

The R package “ConsensusClusterPlus” was used to identify cuproptosis subtypes according to the expression levels of CRGs (29). To confirm variations in the distribution of cuproptosis subtypes, principal component analysis (PCA) was applied. Next, we investigated how cuproptosis subtypes related to prognosis and other clinicopathological characteristics, including age, sex, mechanical ventilation status, diabetic status, and whether admitted to the ICU, in order to evaluate the clinical significance of these two subtypes. We compared the HFD45 values of different cuproptosis subtypes to assess prognostic differences. Box plots were used to compare the CRG expression levels in the two cuproptosis subtypes. Sankey plots were then plotted based on the ggalluvial R package to visualize the relationship between cuproptosis subtypes and other clinical variables. Finally, we mapped the heatmap of gene expression patterns of CRGs in different subgroups.

### Functional enrichment profiling and immunological landscape of cuproptosis subgroups

Gene set variation analysis (GSVA) is an analytical approach for enrichment analysis of microarray and RNA-seq data under parameter-free and unsupervised conditions, which probes the differences in target gene sets across samples by calculating normalized enrichment statistics (NES) (30). The biological functions that differ between cuproptosis clusters were displayed using a heatmap based on the NES of patients with COVID-19. With the help of the limma package, we obtained differentially expressed genes (DEGs) from the two cuproptosis subtypes and carried out gene set enrichment analysis (GSEA). P<0.05 was considered as a statistically significant difference (31, 32). IOBR is an R software package that integrates eight published methods for decoding the immune microenvironment: CIBERSORT, TIMER, xCell, MCPcounter, ESTIMATE, EPIC, IPS, quanTIseq, thus being used to explore the differential profile of immune cell types in different samples (33). The CIBERSORT algorithm was applied to quantify the level of infiltration of 22 immune cell signatures for each COVID-19 sample and the immune score of COVID-19 patients was calculated using the ESTIMATE algorithm (34, 35). To validate the immunological profiles of the cuproptosis subtypes, the differences in gene expression of T cell stimulators and major histocompatibility complexes were compared between different clusters. Subsequently, variations in clinical characteristics were compared between the two clusters. P< 0.05 was considered to be statistically different.

### Construction of the CRRS

In order to gain deeper insights into the underlying molecular mechanisms of the cuproptosis pathway, we conducted a comprehensive screening of potential biomarkers within the GEO cohort. The screening process involved the utilization of LASSO, random forest, and support vector machine algorithms. Initially, these three machine learning algorithms were applied to identify the differentially expressed genes between the two subtypes. Subsequently, these identified genes were employed in the construction of a predictive model. Genes were then included in multivariate logistic regression analysis with p-values less than 0.05 to establish the CRRS based on the regression coefficients. $\text{CRRS}=\sum_{i}^{n}Ci\times Ei$ and n, C, E represent the number of signature CRGs, the coefficients, and the gene expression level, correspondingly. According to the median value of the CRRS, patients were grouped into two groups, the high-risk group and the low-risk group, and the differences in the correlation of the CRRS with clinical indicators between the two groups were compared. The reliability of the CRRS is commonly evaluated using recipient operating characteristic curve (ROC). The pROC package was utilized to perform ROC analysis (36). To assess the effectiveness of the CRRS and compare it with other CRGs, the AUC values of the ROC curves were generated. Meanwhile, to further validate the accuracy of the scoring model, ROC curve analysis was also performed on several external validation sets (GSE152418 and GSE171110).

### Building and assessment of a nomogram

In order to determine if CRRS could be considered as an independent factor for COVID-19 and build the cuproptosis-related model, univariate and multivariate logistic regression analyses were used. Then, a nomogram was constructed by combining age, sex, diabetic status, whether admitted to ICU, mechanical ventilation status, HFD45, Charlson score, and CRRS. The rms package created a calibration plot to demonstrate the consistency between the expected endpoint events and the true outcome. ROC curve was routinely applied to test the reliability of the cuproptosis-related-model.

### Validation of model genes based on proteomics and single cell analysis

Based on the findings from our previously published proteomics studies conducted by our research team, we proceeded to validate the expression levels of the corresponding proteins associated with the four model genes in both COVID-19 tissue samples and control samples (37). Subsequently, we extended our validation efforts by analyzing the expression of these four model genes in COVID-19 using single-cell analysis. This analysis was performed using SPEED, an online single-cell multi-omics analysis tool that incorporates diverse datasets from over 120 species, encompassing evolutionary, developmental, and disease-related information (38). The single-cell sequencing data from five COVID-19 patients can be accessed in the CNGB Nucleotide Sequence Archive (CNSA: https://db.cngb.org/cnsa), with the dataset ID CNP0001102 (39). Initially, we utilized the UMAP algorithm to reduce the dimensionality and cluster the single-cell expression data. Cell types were defined based on classical cell markers. Subsequently, we generated heatmaps to compare the expression levels of the four model genes across different cell types.

## Results

### CRGs are associated with immune characteristics of COVID-19

**Figure 1:**
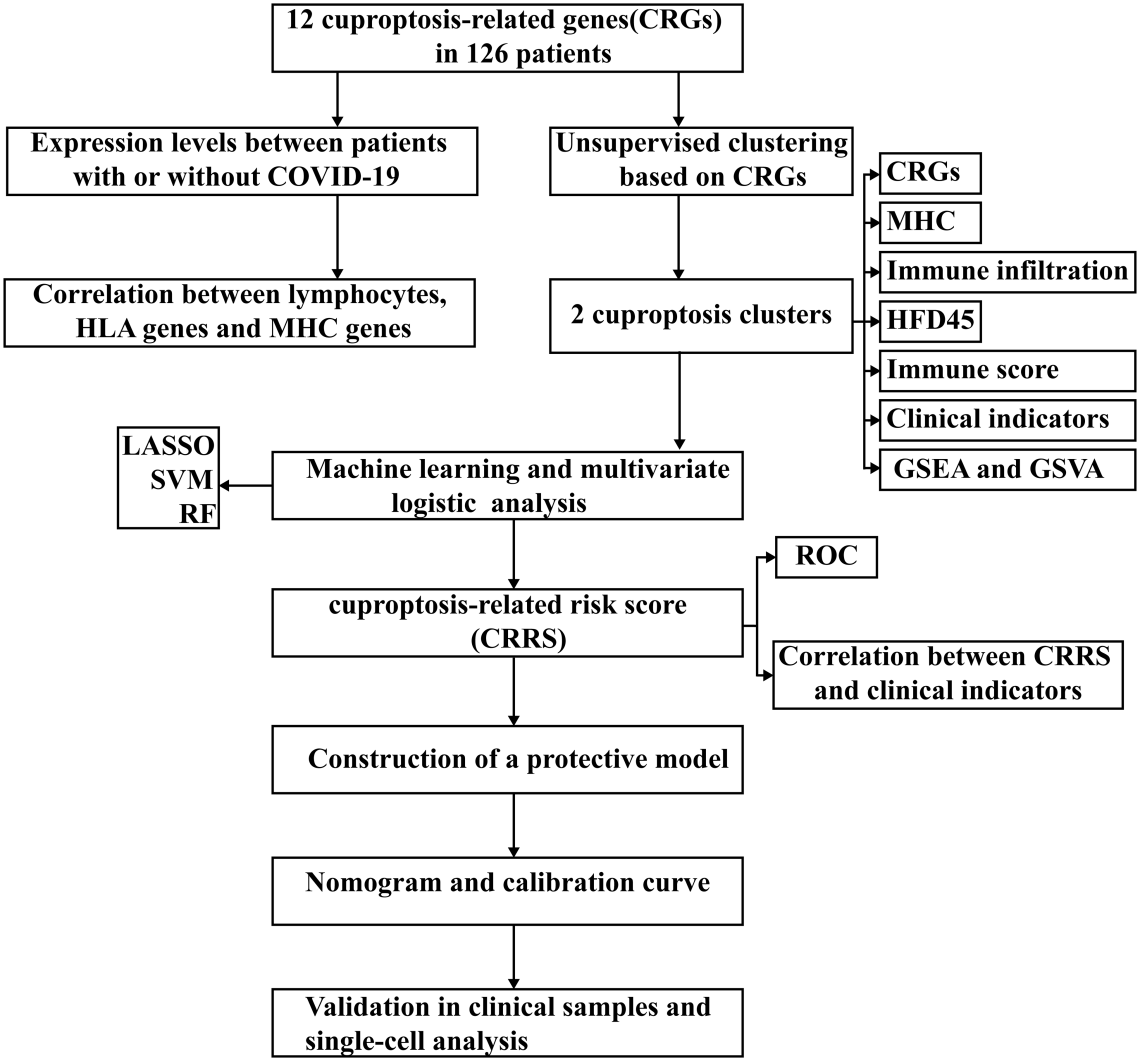
Workflow diagram of this study.

**Figure 2:**
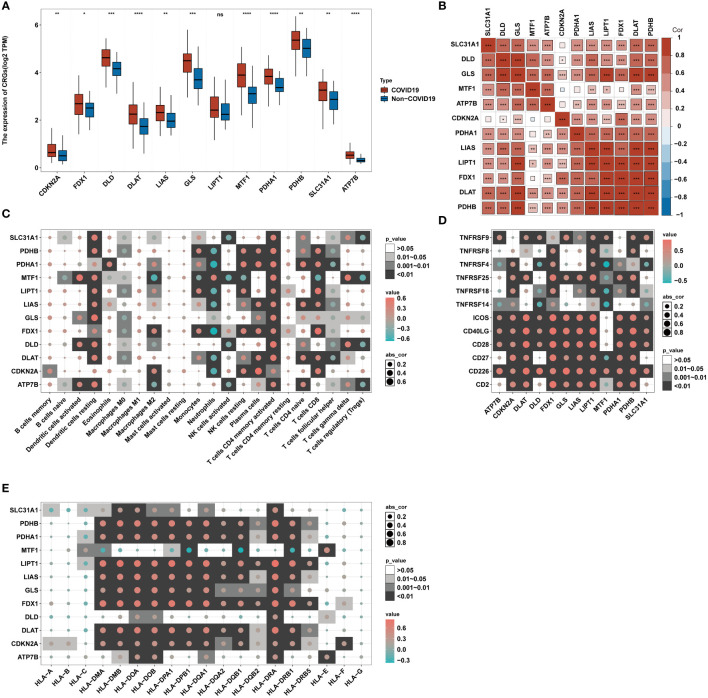
Correlation between CRGs expression and immune characteristics in COVID-19. **(A)** The expression of 12 CRGs of blood leukocytes between patients with or without COVID-19. **(B)** Correlation plot of 12 CRGs. The positive correlation was marked with red, and negative correlation was marked with blue. **(C)** Heatmap of the correlations between 12 CRGs and 22 immunocytes. **(D)** Heatmap of the correlations between 12 CRGs and 12 T-cell stimulators. **(E)** Heatmap of the correlations between 12 CRGs and 19 human leukocyte antigen (HLA) genes. *p< 0.05, **p< 0.01, ***p< 0.001, ****p< 0.0001, ns, no significance.

**Figure 1** presents the workflow. Twelve CRGs (CDKN2A, FDX1, DLD, DLAT, LIAS, GLS, LIPT1, MTF1, PDHA1, PDHB, SLC31A1, and ATP7B) were analyzed in this work. Except for LIPT1, COVID-19 patients had significantly higher expression levels of the other eleven CRGs. (**Figure 2A**). We portrayed the correlation pattern to investigate the relationships between CRGs (**Figure 2B**). Overall, there was a strong correlation between the 12 CRGs. the highest correlation coefficient (coefficient=0.92) was found between PDHB and DLAT (P<0.05), which means they may function synergistically. Meanwhile, the correlation between CDKN2A and the other CRGs was weak.

To further delineate the association between CRGs and immunological characteristics, such as immune cells, T-cell stimulators, and human leukocyte antigen (HLA)genes, we analyzed their correlation. Through the CIBERSORT algorithm, the proportion of 22 immune cells in COVID-19 was assessed (34). Several infiltrating immune cells were associated with CRGs (**Figure 2C**). The strong relations between neutrophils and CRGs and their strongest positive and negative correlations with MTF1 and FDX1, respectively, imply that MTF1 and FDX1 may regulate neutrophil infiltration in COVID-19. Correlation analysis revealed that T-cell stimulators were closely correlated with CRGs (**Figure 2D**), implying that the expression of T-cell stimulators in COVID-19 may be influenced by CRGs. Similar to immune cells, HLA genes showed strong correlations with CRGs (**Figure 2E**). The strongest positive connection, 0.84, was found between HLA-DRA and LIPT1. The most inverse relationship between HLA-DQB1 and MTF1 was observed, having a correlation coefficient of -0.37. These suggest that CRGs may affect HLA gene expression in COVID-19.

### Cuproptosis subgroups in COVID-19

**Figure 3:**
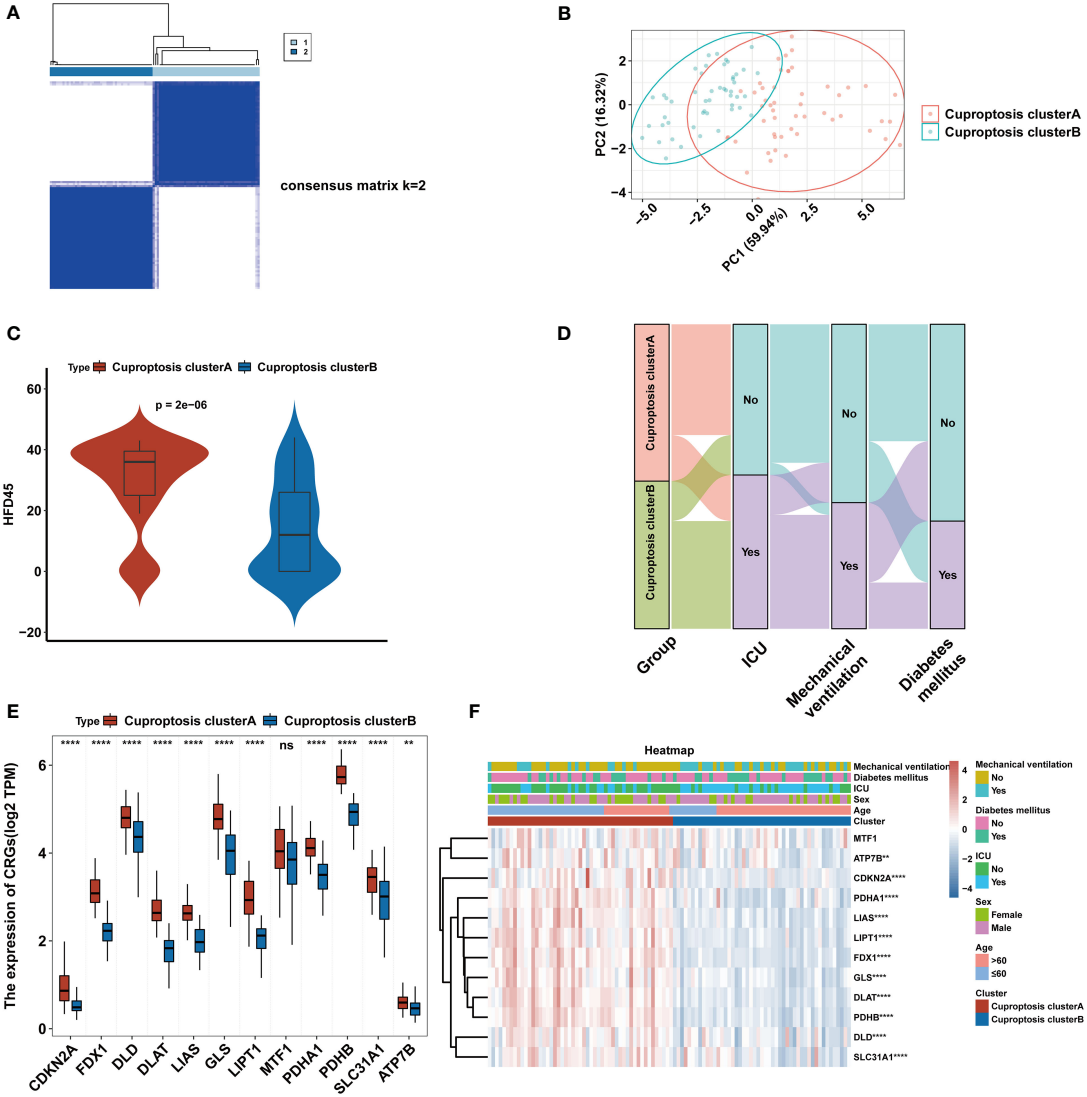
Consensus clustering of CRGs in COVID-19. **(A)** Consensus matrix of patients in the GEO cohort for k = 2. **(B)** PCA analysis of cuproptosis subtypes. **(C)** The HFD45 between the two cuproptosis clusters. **(D)** Alluvial map showing the changes of cuproptosis cluster, ICU status, mechanical ventilation status and diabetes mellitus status. **(E)** The expression of 12 CRGs of blood leukocytes between the two cuproptosis clusters. **(F)** Heatmap of 12 CRGs between the cuproptosis clusters and clinical feature annotation was used. **p < 0.01, ****p < 0.0001, ns, no significance.

In COVID-19, two subtypes were characterized using unsupervised clustering methods, including 51 cases of cuproptosis-related cluster A and 49 cases of cuproptosis-related cluster B (**Figure 3A**). Based on the results of PCA analysis, all patients could be roughly divided into two parts, which further supported that two subtypes are different (**Figure 3B**). Next, we compared the HFD45 values of the two subtypes to evaluate the prognostic disparities between the two clusters (**Figure 3C**). The results showed that HFD45 values were higher for cuproptosis-related cluster A, meaning that cuproptosis-related cluster A had a better prognosis. Next, we investigated how the two groups related to different clinical characteristics (**Figure 3D**). More patients in cluster A were admitted to the ICU, were mechanically ventilated, and suffered from diabetes compared to those in cluster B, which also verified that cluster A had a better prognosis. Except for MTF1, the remaining eleven CRGs were significantly upregulated in cuproptosis-related cluster A (P<0.05) (**Figure 3E**). The transcriptome map of CRGs differentially expressed in the two cuproptosis subtypes was sketched in the heatmap (**Figure 3F**). Based on the analysis of the heatmap, we observed that the CRGs exhibited predominantly high expression levels among female patients who were aged 60 years or younger. Furthermore, this high expression pattern was observed in patients who were not mechanically ventilated and were not admitted to the ICU. However, we did not identify a significant association between the expression of CRGs and the diabetic status of the patients. **Figure S1** shows that ventilator-free days differed between the two subgroups (**Figure S1A**) (P<0.05). In addition, the number of patients admitted to the ICU and undergoing mechanical ventilation also differed between the two subgroups (**Figures S1B, C**) (P<0.05). These suggest that CRGs may influence the development of COVID-19 through several potential mechanisms.

### The immunoscape of cuproptosis subtypes

**Figure 4:**
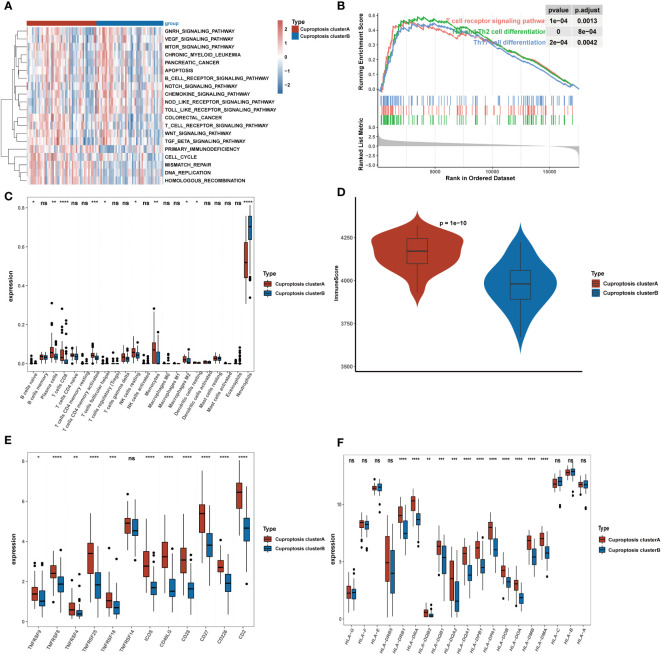
Clinical significance and immune landscape of cuproptosis subtypes in the GEO cohort. **(A)** GSVA analyzed the biological pathways of two cuproptosis subtypes. Red represents the activation of biological pathways and blue represents inhibition of biological pathways. **(B)** Gene set enrichment analysis (GSEA) shows the significant enrichment in immune-associated biological processes. **(C)** The landscape of immune cell infiltration between two cuproptosis subtypes. **(D)** Immune score between two cuproptosis subtypes. **(E)** Gene expression of T-cell stimulators gene sets between two distinct clusters. **(F)** Gene expression of HLA gene sets between two distinct clusters. *p< 0.05, **p< 0.01, ***p< 0.001, ****p< 0.0001, ns, no significance.

We performed GSVA analysis to ascertain discrepancy in enrichment analysis between the two cuproptosis subtypes and discovered that the two subtypes displayed different immune infiltration patterns. The heatmap revealed that immunological pathways, such as T-cell receptor signaling pathways and B-cell receptor signaling pathways, were considerably abundant in cluster A (**Figure 4A**). Meanwhile, GSEA analysis was carried out to verify the variations in immunological pathways in the two cuproptosis clusters. The findings revealed that the T cell receptor signaling pathway, Th1 and Th2 cell differentiation, and Th17 cell differentiation were considerably enriched in DEGs substantially expressed in cluster A (**Figure 4B**). Given the strong correlation between cuproptosis subtypes and immunoreactivity, we used the CIBERSORT algorithm to determine the degree of immune infiltration in both clusters (**Figure 4C**). While the cluster B subtype was distinguished by high infiltration of neutrophils, the cluster A subtype was characterized by high abundance of naive B cells, plasma cells, CD8 T cells, activated memory CD4 cells, follicular helper T cells, resting NK cells, monocytes, macrophages M2, and resting dendritic cells. Then, the ESTIMATE method was used to determine the immune score. As shown in **Figure 4D**, higher immune scores were also exhibited in cluster A compared to cluster B. Finally, we investigated how both subtypes related to HLA genes and T-cell stimulators. T-cell stimulators were higher in cluster A, with the exception of TNFRSF14 (**Figure 4E**). The expression levels of HLA genes tended to be higher in cluster A except for HLA-A, HLA-B, HLA-C, HLA-E, HLA-F, HLA-G, and HLA-DRB5 (**Figure 4F**).

### CRRS for the prediction of COVID-19

**Figure 5:**
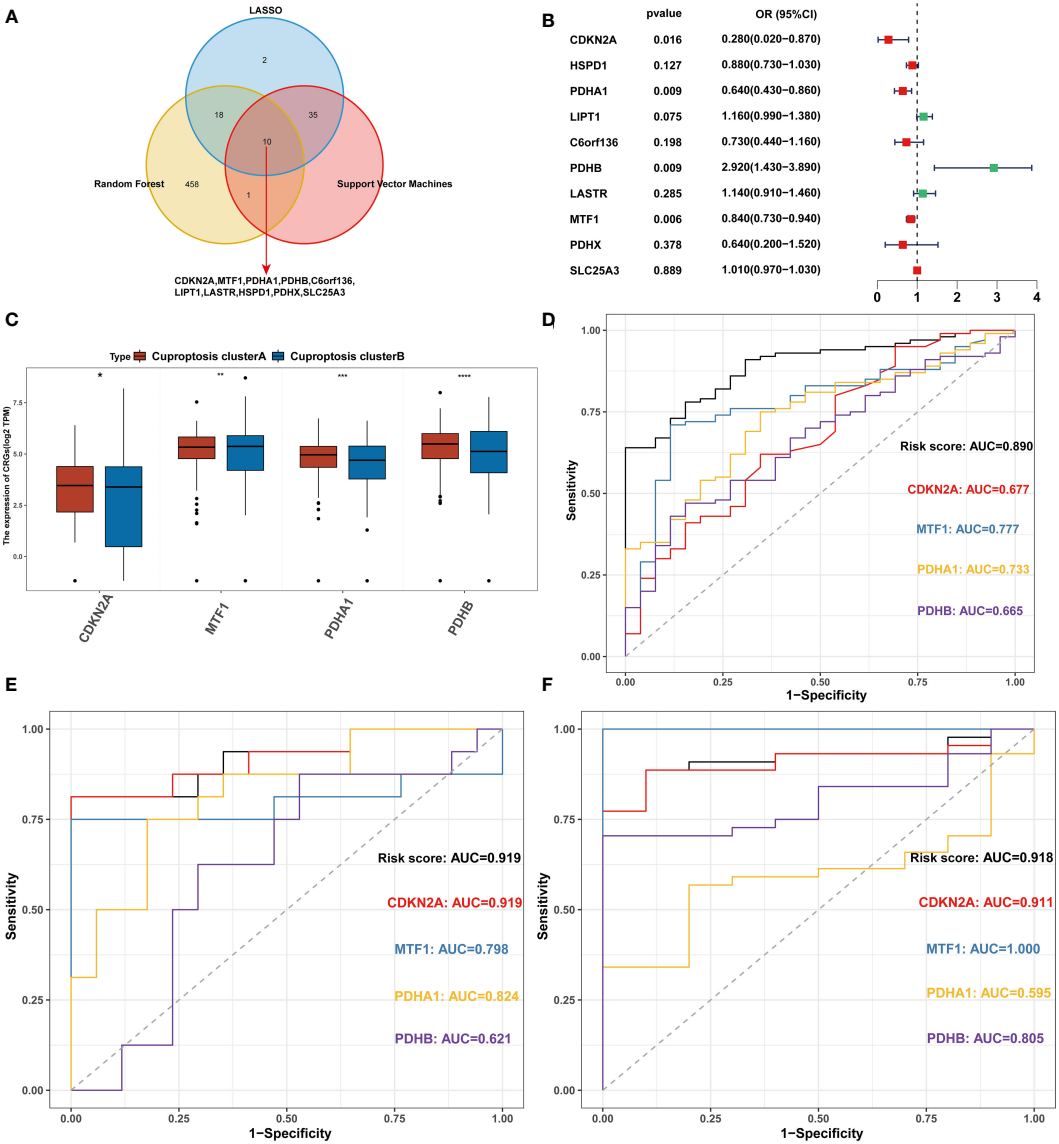
Construction of the CRRS. **(A)** Intersection of critical genes *via* multiple machine-learning algorithms. **(B)** Multivariate analysis for the GEO cohort. **(C)** The expression of 4 signature-related genes between the two cuproptosis clusters. **(D)** ROC analyses of the diagnostic efficacy for the CRRS and 4 signature-related genes in GSE157103. **(E)** ROC analyses of the diagnostic efficacy for the CRRS and 4 signature-related genes in GSE152418. **(F)** ROC analyses of the diagnostic efficacy for the CRRS and 4 signature-related genes in GSE171110. *p< 0.05, **p< 0.01, ***p< 0.001, ****p< 0.0001, ns, no significance.

**Figure 6:**
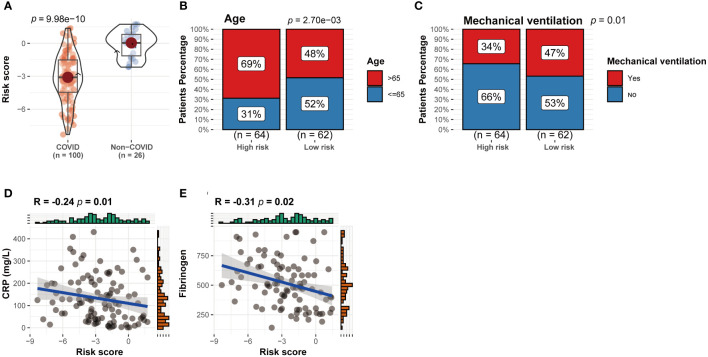
Correlation between CRRS expression and clinical parameters. **(A)** CRRS between the two cuproptosis clusters. **(B–E)** The correlations between CRRS and age **(B)**, the number of patients undergoing mechanical ventilation **(C)**, CRP **(D)** and ferritin **(E)**.

All 126 patients in the GEO cohort were subjected to model construction. First, ten genes were screened based on machine learning algorithms (**Figure 5A**). After including these genes in the multivariate logistic regression model, the expression values of 4 CRGs were used to construct the CRRS signature (**Figure 5B**). The formula for calculating the CRRS was as follows: CRRS= (-3.38548*CDKN2A)+ (-0.30651*MTF1)+ (-0.42584*PDHA1)+ (0.16582*PDHB). The expression values of the four model-related genes were validated using the GSE163151 dataset and they were found to be differentially expressed between the two cuproptosis clusters, which is also consistent with the results in **Figure 3E** (**Figure 5C**). The combined model showed the most significant area under the ROC curve (AUC) compared to other individual models in three separate datasets (GSE157103, GSE152418, and GSE171110), demonstrating the best prediction performance of the combined model (**Figures 5D-F**). Furthermore, the optimal cutoff value of the ROC curve for the risk score was calculated to be -2.172, with a specificity and sensitivity of 1.000 and 0.640 respectively. Consequently, individuals with a risk score lower than -2.172 can be identified as having a COVID-19 infection. In this study, we found that COVID-19 patients have lower risk score values than non-COVID-19 patients, indicating that a lower risk score value is more likely to be diagnosed with COVID-19(**Figure 6A**). The above results further elucidate the fact that only models containing the four CRGs mentioned above can produce accurate prediction.

Patients were grouped into two groups, the high-risk group and the low-risk group, based on the median value of the CRRS. Between the two groups, there were differences in the number of patients receiving mechanical ventilation and the age distribution of the patients (**Figures 6B, C**) (P<0.05). In addition, CRP and ferritin levels were negatively correlated (P<0.05) (**Figures 6D, E**), which implies that CRGs may influence COVID-19 progression through lactate metabolism and ferritin metabolism.

### CRRS can be considered as an independent factor in COVID-19

**Figure 7:**
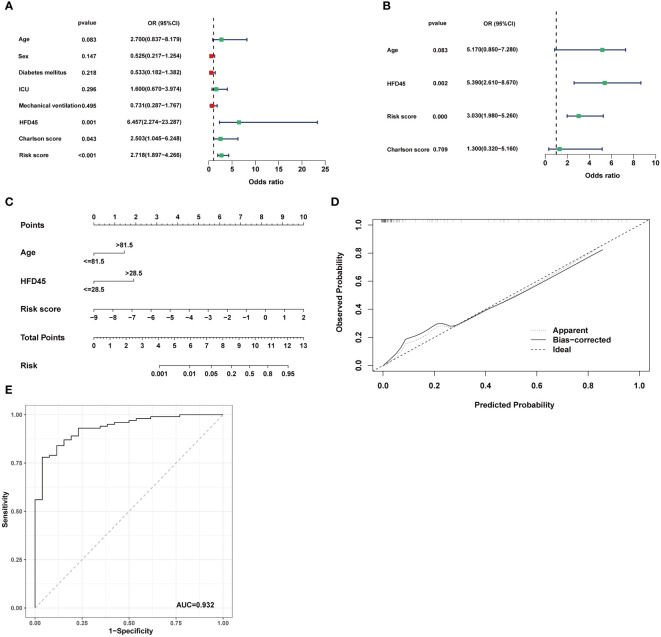
Establishment of the nomogram model. **(A, B)** Univariate analysis and multivariate analysis containing CRRS and clinical factors. **(C)** Establishment of the nomogram model based on age, HFD45 and CRRS. **(D)** Predictive robustness of the nomogram model as disclosed by the calibration curve. **(E)** ROC analysis of the diagnostic efficacy for the nomogram model.

We used logistic regression analysis to examine if CRRS was a independent factor in COVID-19. Age, sex, diabetic status, whether admitted to ICU, mechanical ventilation status, HFD45, Charlson score, and risk score were analyzed as covariates. The findings demonstrated that the independent predictors of COVID-19 occurrence were HFD45, risk score, and Charlson score (**Figures 7A, B**). Since age is a crucial determinant of COVID-19 severity and progression (40), by combining independent factors and age, we created a nomogram, as a therapeutically useful quantitative technique to estimate the likelihood of prevalence in COVID-19 patients (**Figure 7C**). Additionally, the calibration plot demonstrated that the nomogram’s performance was comparable to that of the ideal model (**Figure 7D**). The nomogram displayed strong predictive power, as demonstrated by the ROC (**Figure 7E**).

### Verification of the model genes in clinical samples and single-cell analysis

**Figure 8:**
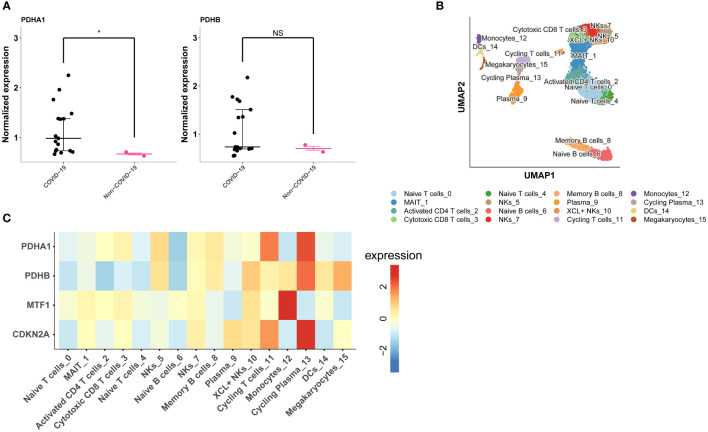
Validation of model genes expression in COVID-19 lung tissue proteomics and single-cell data. **(A)** PDHA1 and PDHB expression in COVID-19 lung tissue proteomics. **(B)** The UMAP plot shows the cell types identified in COVID-19 with different colors. **(C)** Heatmap depicts PDHA1, PDHB, MTF1 and CDKN2A expression across major cell types. *p< 0.05, **p< 0.01, ***p< 0.001, ****p< 0.0001, NS, no significance.

Among the four model genes, we observed the detection of corresponding proteins only for PDHA1 and PDHB. In COVID-19 samples, the expression of PDHA1 was significantly higher compared to control samples, while the difference in PDHB expression between COVID-19 and control samples was not statistically significant (**Figure 8A**). Subsequently, we explored the relationship between the expression of the four model genes and different cell populations. Through clustering, we identified 16 distinct cell clusters, which were further consolidated into 14 cell populations based on marker gene expression. These populations included MAIT cells, Activated CD4 T cells, Cytotoxic CD8 T cells, Naive T cells, Naive B cells, NK cells, Memory B cells, Plasma XCL+ NK cells, Cycling T cells, Monocytes, Cycling Plasma cells, Dendritic cells (DCs), and Megakaryocytes (**Figure 8B**). Our findings revealed that PDHA1, PDHB, and CDKN2A exhibited prominent expression in Cycling Plasma cells, while MTF1 demonstrated predominant expression in Monocytes (**Figure 8C**).

## Discussion

Patients with COVID-19 may experience immune system changes. Tian et al. discovered that the number of helper T (Th) cells and Tregs in COVID-19 were below normal levels, which are both more obviously decreased in severe groups (21). In addition, the expression of inhibitory receptors on CD8+ T cells, including PD-1, TIM-3, TIGIT, CTLA-4, and NKG2A were increased in the early phase after infection (41). Moreover, the magnitude of anti-SARS-CoV-2 antibody reaction correlated with the severity of COVID-19 disease. It has been demonstrated that T-cell memory unique to SARS-CoV-2 can be preserved for ten months in patients convalescing from COVID-19 (42, 43). However, the fundamental mechanism of immune cell activation in COVID-19 is still not fully understood.

As an essential trace element in the body, copper has a broad and vital role in biological systems. Copper metabolism in the body is in a state of dynamic equilibrium, termed copper homeostasis. When copper homeostasis in the body is disrupted, abnormal copper metabolism can lead to a series of diseases. Diseases such as Wilson′s disease, Menkes disease, Alzheimer′s disease, Parkinson′s disease, obesity, hypertension, and tumors have been proven to be implicated in abnormal copper metabolism (44–50). Copper is engaged in the functionality of immune cells such as natural killer cells and macrophages, based on which it can help kill some viruses such as bronchitis viruses, single- or double-stranded DNA, and RNA viruses (51). Interestingly, copper also exerts a role in COVID-19. It was found that whole blood copper levels were significantly higher in COVID-19 patients with severe condition compared to those with non-severe condition (52). In addition, during the initial stages of the disease, COVID-19 patients had increased serum levels of copper ions, which were mainly associated with the inflammatory response (53). Moreover, copper ion levels are valuable in the prognosis prediction of COVID-19 patients. One research found that serum copper and selenium levels in COVID-19 patients helped predict patient prognosis, and copper supplementation in patients diagnosed with copper deficiency may improve the prognosis of the disease (54). Cuproptosis, as a newly observed copper ion-induced cell death form, copper death, has been demonstrated to be a potential therapeutic target for Wilson’s disease and cancer, but its role in COVID-19 remains unclear (55).

In this study, we performed a thorough analysis of the cuproptosis landscape in COVID-19 patients. When compared to those who did not have COVID-19, the expression levels of CRGs were higher in COVID-19 patients’ blood lymphocytes, indicating that cuproptosis may play an essential role in COVID-19 patients.

Subsequently, based on the expression of CRGs, we discovered that COVID-19 could be divided into two subgroups, cuproptosis cluster A and cuproptosis cluster B. These two subtypes showed a significantly different prognosis, with cluster A having higher HFD45 values than cluster B. More patients in cluster B had diabetes, and had been treated by mechanical ventilation, as revealed by an analysis of clinical features, helps to explain why this cluster has a worse survival rate. The causes of these disparities were clarified using GSVA and GSEA enrichment analysis. The findings demonstrated that T cell receptor signaling, Th1 and Th2 cell differentiation, and Th17 cell differentiation were highly enriched in cluster A, which is associated with immunological activation. Therefore, we looked into the connection between immune cell infiltration and two cuproptosis subtypes.

Because of the complexity of the human immune system, various immune cells have various functions. Macrophages typically consist of two subtypes, with M1 macrophages playing a pro-inflammatory role and M2 macrophages playing an anti-inflammatory and immunomodulatory role by secreting IL-10 and TGF-β to assist in tissue repair, revascularization, and homeostasis maintenance while reducing inflammation (56). In humans, neutrophils are the most prevalent immune cells. Previous studies have highlighted the potential association between elevated neutrophil levels and unfavorable tumor prognosis. This correlation can be attributed to several factors, including the immunosuppressive effects of neutrophils, their ability to promote tumor growth, and their facilitation of tumor cell migration and invasion through the release of factors such as hepatocyte growth factor (HGF) (57–59). In renal cell carcinoma, intratumoral neutrophils, along with other factors like myeloid-derived suppressor cells, arginase, reactive oxygen species, B7-Hx, and PD-1, contribute to the inhibition of an effective immune response, thus allowing the tumor to evade immune surveillance and foster its growth (59). Similarly, in bronchioloalveolar carcinoma, tumor-infiltrating neutrophils produce HGF, which promotes the migration of tumor cells through its interaction with the c-met receptor on tumor cells. Elevated levels of HGF in bronchoalveolar lavage fluid are associated with poorer clinical outcomes in patients with bronchioloalveolar carcinoma (58). In the present study, we observed that cuproptosis cluster A exhibited lower neutrophil infiltration compared to cuproptosis cluster B. Furthermore, cuproptosis cluster A demonstrated a higher HFD45 value, providing further validation that cuproptosis cluster A is associated with a more favorable prognosis. The conversion of B cells into plasma cells for antibody production is facilitated by T-follicular helper cells, which is essential for eradicating viruses and bacteria. Several studies have demonstrated that T-follicular helper cells help to contain hepatitis C virus infection, human immunodeficiency virus infection, and group A streptococcal bacterial infection (60). CD8+ T cells are cytotoxic cells. The presence of virus-specific CD8+ T lymphocytes was linked to better COVID-19 outcomes in SARS-CoV-2 infection (61). Natural killer cells are important early effector lymphocytes. Lower NK cell counts have been reported to be associated with poorer survival rates in COVID-19 (62). As previously described, we determined the relationship between cuproptosis and T-cell activators, HLA genes, and immune cell infiltration and found that cuproptosis and immune regulation are tightly linked. Next, we examined the connection between immune cell infiltration and cuproptosis subtypes. We found that M2 macrophages, T follicular helper cells, CD8 T cells, and natural killer infiltrated more in cluster A than in cluster B, which was associated with anti-inflammatory and viral clearance, and therefore led to a better prognosis. In addition, T-cell stimulators, and human leukocyte antigen (HLA) genes were also upregulated in cluster A, further confirming the better prognosis of cluster A. On the other hand, neutrophils were more infiltrated in cluster B than cluster A, thus leading to a poorer prognosis.

Given the influence of CRGs and cuproptosis subtypes on clinical results, we constructed a cuproptosis-related risk score based on univariate and multifactorial logistic regression analysis using four identified genes (CDKN2A, MTF1, PDHA1, PDHB). CDKN2A is an essential tumor suppressor gene encoding p14ARF and p16INK4A. Lungs of patients who died from SARS-CoV-2 have been reported to express more p16INK4A than those who died from other causes, which may be due to upregulation of CDKN2A leading to cell cycle arrest and thus apoptosis (63, 64). MTF-1 is a zinc-dependent transcription factor that is involved in maintaining intracellular metal homeostasis as well as regulating inflammatory responses. When inflammation occurs, zinc ions are released from metallothioneins. Free zinc ions stimulate MTF-1 function and decrease gene expression of pro-inflammatory cytokines, thereby regulating inflammation (65, 66). PDHA1 encodes the pyruvate dehydrogenase alpha subunit, which is part of the pyruvate dehydrogenase (PDH) complex. PDHA1 has been reported to facilitate the activation of the NLRP3 inflammasome in response to COVID-19 infection (67). Similar to PDHA1, PDHB encodes the pyruvate dehydrogenase beta subunit (68). PDHB is aberrantly expressed in gastric cancer and is associated with a better prognosis. In addition, it has been shown that inhibition of PDHB promotes colorectal cancer growth and metastasis (69, 70). However, there is no report of PDHB with COVID-19.

In this study, the CRRS achieved high AUC values in several datasets. We conducted a comparative analysis of the CRRS with existing models for COVID-19 prediction, and the results demonstrated the excellent predictive performance of the CRRS. Sun et al. developed a model utilizing ferroptotic genes, achieving an AUC value of 0.897 (71). Zhou et al. employed machine learning methods to construct a disease diagnostic model with an AUC value of 0.815 (72). Moreover, Nguyen et al. introduced a novel index, CD24-CSF1R, which exhibited a significant correlation with COVID-19 severity, yielding an AUC of 0.850 (73). These findings indicate that the CRRS demonstrates competitive discrimination power and model performance, as it achieves comparable or even higher AUC values when compared to the referenced models. What’s more, patients with COVID-19 had a lower risk score than those with COVID-19; thus, the CRRS is a protective score. Previous studies have reported that CRP and fibrinogen were more elevated in patients with COVID-19 (74, 75). Coincidentally, the findings of correlation analysis revealed that the CRRS was adversely linked to CRP and fibrinogen, consistent with the results of the study as mentioned above. These findings suggest that CRRS is a promising predictor of clinical result and prognosis in patients with COVID-19. Finally, combined with other clinical parameters, univariate and multifactorial logistic regression analyses showed that CRRS was an independent factor for COVID-19.

This study possesses certain limitations that warrant acknowledgment. Firstly, the precise relationship between CRGs and COVID-19 necessitates further evaluation and validation in larger sample sizes and diverse populations. In addition, we could not analyze the precise prognostic value of CRRS because HFD45 only provided a rough prognostic response, and the GSE157103 dataset did not provide specific survival information.

In conclusion, our investigation showed that patients with different cuproptosis subtypes had different immune infiltration features. The CRRS can reliably identify patients contracting COVID-19 and predict clinical results. In conclusion, our research sheds new light on cuproptosis in SARS-CoV-2-infected patients’ blood cells. It provides a tool for assessing clinical prognosis and the likelihood of COVID-19 infection.

## Data availability statement

The original contributions presented in the study are included in the article/**Supplementary Material**. Further inquiries can be directed to the corresponding author.

## Author contributions

HL and JSY performed data analyses and wrote the manuscript draft. DYZ and XZ revised the manuscript. All authors read and approved the final manuscript.

## References

[1] Sakurai T Kataoka K. Structure and function of type I copper in multicopper oxidases. Cell Mol Life Sci (2007) 64(19-20):2642–56. doi: 10.1007/s00018-007-7183-y PMC11136192 17639274

[2] Kahlson MA Dixon SJ. Copper-induced cell death. Science (2022) 375(6586):1231–2. doi: 10.1126/science.abo3959 35298241

[3] Ren Y Shu T Wu D Mu J Wang C Huang M. The ORF3a protein of SARS-CoV-2 induces apoptosis in cells. Cell Mol Immunol (2020) 17(8):881–3. doi: 10.1038/s41423-020-0485-9 PMC7301057 32555321

[4] Thompson EA Cascino K Ordonez AA Zhou W Vaghasia A Hamacher-Brady A. Metabolic programs define dysfunctional immune responses in severe COVID-19 patients. Cell Rep (2021) 34(11):108863. doi: 10.1016/j.celrep.2021.108863 33691089 PMC7908880

[5] Bellesi S Metafuni E Hohaus S Maiolo E Marchionni F D'Innocenzo S. Increased CD95 (Fas) and PD-1 expression in peripheral blood T lymphocytes in COVID-19 patients. Br J Haematol (2020) 191(2):207–11. doi: 10.1111/bjh.17034 PMC7405050 32679621

[6] Li S Zhang Y Guan Z Li H Ye M Chen X. SARS-CoV-2 triggers inflammatory responses and cell death through caspase-8 activation. Signal Transduct Target Ther (2020) 5(1):235. doi: 10.1038/s41392-020-00334-0 33037188 PMC7545816

[7] Feoktistova M Geserick P Kellert B Dimitrova DP Langlais C Hupe M. cIAPs block ripoptosome formation, a RIP1/caspase-8 containing intracellular cell death complex differentially regulated by cFLIP isoforms. Mol Cell (2011) 43(3):449–63. doi: 10.1016/j.molcel.2011.06.011 PMC3163271 21737330

[8] Bertheloot D Latz E Franklin BS. Necroptosis, pyroptosis and apoptosis: an intricate game of cell death. Cell Mol Immunol (2021) 18(5):1106–21. doi: 10.1038/s41423-020-00630-3 PMC8008022 33785842

[9] Chen IY Moriyama M Chang MF Ichinohe T. Severe acute respiratory syndrome coronavirus viroporin 3a activates the NLRP3 inflammasome. Front Microbiol (2019) 10:50. doi: 10.3389/fmicb.2019.00050 30761102 PMC6361828

[10] Murakami T Ockinger J Yu J Byles V McColl A Hofer AM. Critical role for calcium mobilization in activation of the NLRP3 inflammasome. Proc Natl Acad Sci U.S.A. (2012) 109(28):11282–7. doi: 10.1073/pnas.1117765109 PMC3396518 22733741

[11] DeDiego ML Nieto-Torres JL Regla-Nava JA Jimenez-Guardeño JM Fernandez-Delgado R Fett C. Inhibition of NF-κB-mediated inflammation in severe acute respiratory syndrome coronavirus-infected mice increases survival. J Virol (2014) 88(2):913–24. doi: 10.1128/JVI.02576-13 PMC3911641 24198408

[12] Siu KL Yuen KS Castaño-Rodriguez C Ye ZW Yeung ML Fung SY. Severe acute respiratory syndrome coronavirus ORF3a protein activates the NLRP3 inflammasome by promoting TRAF3-dependent ubiquitination of ASC. FASEB J (2019) 33(8):8865–77. doi: 10.1096/fj.201802418R PMC6662968 31034780

[13] Yap JKY Moriyama M Iwasaki A. Inflammasomes and pyroptosis as therapeutic targets for COVID-19. J Immunol (2020) 205(2):307–12. doi: 10.4049/jimmunol.2000513 PMC7343621 32493814

[14] Li Q Chen Z Zhou X Li G Zhang C Yang Y. Ferroptosis and multi-organ complications in COVID-19: mechanisms and potential therapies. Front Genet (2023) 14:1187985. doi: 10.3389/fgene.2023.1187985 37303950 PMC10250669

[15] Tsvetkov P Coy S Petrova B Dreishpoon M Verma A Abdusamad M. Copper induces cell death by targeting lipoylated TCA cycle proteins. Science (2022) 375(6586):1254–61. doi: 10.1126/science.abf0529 PMC9273333 35298263

[16] Tan HY Wang N Zhang C Chan YT Yuen MF Feng Y. Lysyl oxidase-like 4 fosters an immunosuppressive microenvironment during hepatocarcinogenesis. Hepatology (2021) 73(6):2326–41. doi: 10.1002/hep.31600 PMC8251926 33068461

[17] Voli F Valli E Lerra L Kimpton K Saletta F Giorgi FM. Intratumoral copper modulates PD-L1 expression and influences tumor immune evasion. Cancer Res (2020) 80(19):4129–44. doi: 10.1158/0008-5472.CAN-20-0471 32816860

[18] Choi BY Jang BG Kim JH Seo JN Wu G Sohn M. Copper/zinc chelation by clioquinol reduces spinal cord white matter damage and behavioral deficits in a murine MOG-induced multiple sclerosis model. Neurobiol Dis (2013) 54:382–91. doi: 10.1016/j.nbd.2013.01.012 23360710

[19] Huang C Wang Y Li X Ren L Zhao J Hu Y. Clinical features of patients infected with 2019 novel coronavirus in Wuhan, China. Lancet (2020) 395(10223):497–506. doi: 10.1016/S0140-6736(20)30183-5 31986264 PMC7159299

[20] Wang F Nie J Wang H Zhao Q Xiong Y Deng L. Characteristics of peripheral lymphocyte subset alteration in COVID-19 pneumonia. J Infect Dis (2020) 221(11):1762–9. doi: 10.1093/infdis/jiaa150 PMC7184346 32227123

[21] Qin C Zhou L Hu Z Zhang S Yang S Tao Y. Dysregulation of immune response in patients with coronavirus 2019 (COVID-19) in Wuhan, China. Clin Infect Dis (2020) 71(15):762–8. doi: 10.1093/cid/ciaa248 PMC7108125 32161940

[22] Rodrigues TS de Sá KSG Ishimoto AY Becerra A Oliveira S Almeida L. Inflammasomes are activated in response to SARS-CoV-2 infection and are associated with COVID-19 severity in patients. J Exp Med (2021) 218(3):e20201707. doi: 10.1084/jem.20201707 33231615 PMC7684031

[23] Li X Zhang Z Wang Z Gutiérrez-Castrellón P Shi H. Cell deaths: involvement in the pathogenesis and intervention therapy of COVID-19. Signal Transduct Target Ther (2022) 7(1):186. doi: 10.1038/s41392-022-01043-6 35697684 PMC9189267

[24] Overmyer KA Shishkova E Miller IJ Balnis J Bernstein MN Peters-Clarke TM. Large-Scale multi-omic analysis of COVID-19 severity. Cell Syst (2021) 12(1):23–40.e7. doi: 10.1016/j.cels.2020.10.003 33096026 PMC7543711

[25] Qiu X Hua X Li Q Zhou Q Chen J. m(6)A regulator-mediated methylation modification patterns and characteristics of immunity in blood leukocytes of COVID-19 patients. Front Immunol (2021) 12:774776. doi: 10.3389/fimmu.2021.774776 34917088 PMC8669770

[26] Ng DL Granados AC Santos YA Servellita V Goldgof GM Meydan C. A diagnostic host response biosignature for COVID-19 from RNA profiling of nasal swabs and blood. Sci Adv (2021) 7(6):eabe5984. doi: 10.1126/sciadv.abe5984 33536218 PMC7857687

[27] Arunachalam PS Wimmers F Mok CKP Perera R Scott M Hagan T. Systems biological assessment of immunity to mild versus severe COVID-19 infection in humans. Science (2020) 369(6508):1210–20. doi: 10.1126/science.abc6261 PMC7665312 32788292

[28] Lévy Y Wiedemann A Hejblum BP Durand M Lefebvre C Surénaud M. CD177, a specific marker of neutrophil activation, is associated with coronavirus disease 2019 severity and death. iScience (2021) 24(7):102711. doi: 10.1016/j.isci.2021.102711 34127958 PMC8189740

[29] Wilkerson MD Hayes DN. ConsensusClusterPlus: a class discovery tool with confidence assessments and item tracking. Bioinformatics (2010) 26(12):1572–3. doi: 10.1093/bioinformatics/btq170 PMC2881355 20427518

[30] Hänzelmann S Castelo R Guinney J. GSVA: gene set variation analysis for microarray and RNA-seq data. BMC Bioinf (2013) 14:7. doi: 10.1186/1471-2105-14-7 PMC3618321 23323831

[31] Subramanian A Tamayo P Mootha VK Mukherjee S Ebert BL Gillette MA. Gene set enrichment analysis: a knowledge-based approach for interpreting genome-wide expression profiles. Proc Natl Acad Sci U.S.A. (2005) 102(43):15545–50. doi: 10.1073/pnas.0506580102 PMC1239896 16199517

[32] Ritchie ME Phipson B Wu D Hu Y Law CW Shi W. Limma powers differential expression analyses for RNA-sequencing and microarray studies. Nucleic Acids Res (2015) 43(7):e47. doi: 10.1093/nar/gkv007 25605792 PMC4402510

[33] Zeng D Ye Z Shen R Yu G Wu J Xiong Y. IOBR: multi-omics immuno-oncology biological research to decode tumor microenvironment and signatures. Front Immunol (2021) 12:687975. doi: 10.3389/fimmu.2021.687975 34276676 PMC8283787

[34] Newman AM Liu CL Green MR Gentles AJ Feng W Xu Y. Robust enumeration of cell subsets from tissue expression profiles. Nat Methods (2015) 12(5):453–7. doi: 10.1038/nmeth.3337 PMC4739640 25822800

[35] Yoshihara K Shahmoradgoli M Martínez E Vegesna R Kim H Torres-Garcia W. Inferring tumour purity and stromal and immune cell admixture from expression data. Nat Commun (2013) 4:2612. doi: 10.1038/ncomms3612 24113773 PMC3826632

[36] Robin X Turck N Hainard A Tiberti N Lisacek F Sanchez JC. pROC: an open-source package for r and s+ to analyze and compare ROC curves. BMC Bioinf (2011) 12:77. doi: 10.1186/1471-2105-12-77 PMC3068975 21414208

[37] Shu T Ning W Wu D Xu J Han Q Huang M. Plasma proteomics identify biomarkers and pathogenesis of COVID-19. Immunity (2020) 53(5):1108–1122.e5. doi: 10.1016/j.immuni.2020.10.008 33128875 PMC7574896

[38] Chen Y Zhang X Peng X Jin Y Ding P Xiao J. SPEED: single-cell pan-species atlas in the light of ecology and evolution for development and diseases. Nucleic Acids Res (2023) 51(D1):D1150–d1159. doi: 10.1093/nar/gkac930 36305818 PMC9825432

[39] Zhu L Yang P Zhao Y Zhuang Z Wang Z Song R. Single-cell sequencing of peripheral mononuclear cells reveals distinct immune response landscapes of COVID-19 and influenza patients. Immunity (2020) 53(3):685–696.e3. doi: 10.1016/j.immuni.2020.07.009 32783921 PMC7368915

[40] Chen Y Klein SL Garibaldi BT Li H Wu C Osevala NM. Aging in COVID-19: vulnerability, immunity and intervention. Ageing Res Rev (2021) 65:101205. doi: 10.1016/j.arr.2020.101205 33137510 PMC7604159

[41] DiPiazza AT Graham BS Ruckwardt TJ. T Cell immunity to SARS-CoV-2 following natural infection and vaccination. Biochem Biophys Res Commun (2021) 538:211–7. doi: 10.1016/j.bbrc.2020.10.060 PMC7584424 33190827

[42] Jung JH Rha MS Sa M Choi HK Jeon JH Seok H. SARS-CoV-2-specific T cell memory is sustained in COVID-19 convalescent patients for 10 months with successful development of stem cell-like memory T cells. Nat Commun (2021) 12(1):4043. doi: 10.1038/s41467-021-24377-1 34193870 PMC8245549

[43] Röltgen K Boyd SD. Antibody and b cell responses to SARS-CoV-2 infection and vaccination. Cell Host Microbe (2021) 29(7):1063–75. doi: 10.1016/j.chom.2021.06.009 PMC8233571 34174992

[44] Bandmann O Weiss KH Kaler SG. Wilson's disease and other neurological copper disorders. Lancet Neurol (2015) 14(1):103–13. doi: 10.1016/S1474-4422(14)70190-5 PMC4336199 25496901

[45] Tümer Z Møller LB. Menkes disease. Eur J Hum Genet (2010) 18(5):511–8. doi: 10.1038/ejhg.2009.187 PMC2987322 19888294

[46] Sensi SL Granzotto A Siotto M Squitti R. Copper and zinc dysregulation in alzheimer's disease. Trends Pharmacol Sci (2018) 39(12):1049–63. doi: 10.1016/j.tips.2018.10.001 30352697

[47] Bjorklund G Stejskal V Urbina MA Dadar M Chirumbolo S Mutter J. Metals and parkinson's disease: mechanisms and biochemical processes. Curr Med Chem (2018) 25(19):2198–214. doi: 10.2174/0929867325666171129124616 29189118

[48] Gu K Li X Xiang W Jiang X. The relationship between serum copper and Overweight/Obesity: a meta-analysis. Biol Trace Elem Res (2020) 194(2):336–47. doi: 10.1007/s12011-019-01803-6 31300957

[49] He P Li H Liu C Liu M Zhang Z Zhang Y. U-Shaped association between dietary copper intake and new-onset hypertension. Clin Nutr (2022) 41(2):536–42. doi: 10.1016/j.clnu.2021.12.037 35030528

[50] Ge EJ Bush AI Casini A Cobine PA Cross JR DeNicola GM. Connecting copper and cancer: from transition metal signalling to metalloplasia. Nat Rev Cancer (2022) 22(2):102–13. doi: 10.1038/s41568-021-00417-2 PMC8810673 34764459

[51] Raha S Mallick R Basak S Duttaroy AK. Is copper beneficial for COVID-19 patients? Med Hypotheses (2020) 142:109814. doi: 10.1016/j.mehy.2020.109814 32388476 PMC7199671

[52] Zeng HL Yang Q Yuan P Wang X Cheng L. Associations of essential and toxic metals/metalloids in whole blood with both disease severity and mortality in patients with COVID-19. FASEB J (2021) 35(3):e21392. doi: 10.1096/fj.202002346RR 33577131 PMC7995111

[53] Ivanova ID Pal A Simonelli I Atanasova B Ventriglia M Rongioletti M. Evaluation of zinc, copper, and Cu:Zn ratio in serum, and their implications in the course of COVID-19. J Trace Elem Med Biol (2022) 71:126944. doi: 10.1016/j.jtemb.2022.126944 35168023 PMC8820953

[54] Hackler J Heller RA Sun Q Schwarzer M Diegmann J Bachmann M. Relation of serum copper status to survival in COVID-19. Nutrients (2021) 13(6):1898. doi: 10.3390/nu13061898 34072977 PMC8229409

[55] Chen L Min J Wang F. Copper homeostasis and cuproptosis in health and disease. Signal Transduct Target Ther (2022) 7(1):378. doi: 10.1038/s41392-022-01229-y 36414625 PMC9681860

[56] Shapouri-Moghaddam A Mohammadian S Vazini H Taghadosi M Esmaeili SA Mardani F. Macrophage plasticity, polarization, and function in health and disease. J Cell Physiol (2018) 233(9):6425–40. doi: 10.1002/jcp.26429 PMC13482560 29319160

[57] Cupp MA Cariolou M Tzoulaki I Aune D Evangelou E Berlanga-Taylor AJ. Neutrophil to lymphocyte ratio and cancer prognosis: an umbrella review of systematic reviews and meta-analyses of observational studies. BMC Med (2020) 18(1):360. doi: 10.1186/s12916-020-01817-1 33213430 PMC7678319

[58] Wislez M Rabbe N Marchal J Milleron B Crestani B Mayaud C. Hepatocyte growth factor production by neutrophils infiltrating bronchioloalveolar subtype pulmonary adenocarcinoma: role in tumor progression and death. Cancer Res (2003) 63(6):1405–12. 12649206

[59] Jensen HK Donskov F Marcussen N Nordsmark M Lundbeck F von der Maase H. Presence of intratumoral neutrophils is an independent prognostic factor in localized renal cell carcinoma. J Clin Oncol (2009) 27(28):4709–17. doi: 10.1200/JCO.2008.18.9498 19720929

[60] Dong L He Y Cao Y Wang Y Jia A Wang Y. Functional differentiation and regulation of follicular T helper cells in inflammation and autoimmunity. Immunology (2021) 163(1):19–32. doi: 10.1111/imm.13282 33128768 PMC8044332

[61] Sette A Crotty S. Adaptive immunity to SARS-CoV-2 and COVID-19. Cell (2021) 184(4):861–80. doi: 10.1016/j.cell.2021.01.007 PMC7803150 33497610

[62] Bao C Tao X Cui W Hao Y Zheng S Yi B. Natural killer cells associated with SARS-CoV-2 viral RNA shedding, antibody response and mortality in COVID-19 patients. Exp Hematol Oncol (2021) 10(1):5. doi: 10.1186/s40164-021-00199-1 33504359 PMC7839286

[63] Valeri A Chiricosta L Calcaterra V Biasin M Cappelletti G Carelli S. Transcriptomic analysis of HCN-2 cells suggests connection among oxidative stress, senescence, and neuron death after SARS-CoV-2 infection. Cells (2021) 10(9):2189. doi: 10.3390/cells10092189 34571838 PMC8472605

[64] Tripathi U Nchioua R Prata L Zhu Y Gerdes EOW Giorgadze N. SARS-CoV-2 causes senescence in human cells and exacerbates the senescence-associated secretory phenotype through TLR-3. Aging (Albany NY) (2021) 13(18):21838–54. doi: 10.18632/aging.203560 PMC8507266 34531331

[65] Grzywacz A Gdula-Argasińska J Muszyńska B Tyszka-Czochara M Librowski T Opoka W. Metal responsive transcription factor 1 (MTF-1) regulates zinc dependent cellular processes at the molecular level. Acta Biochim Pol (2015) 62(3):491–8. doi: 10.18388/abp.2015_1038 26336656

[66] Majumder S Ghoshal K Summers D Bai S Datta J Jacob ST. Chromium(VI) down-regulates heavy metal-induced metallothionein gene transcription by modifying transactivation potential of the key transcription factor, metal-responsive transcription factor 1. J Biol Chem (2003) 278(28):26216–26. doi: 10.1074/jbc.M302887200 PMC2365495 12716893

[67] Lin HC Chen YJ Wei YH Lin HA Chen CC Liu TF. Lactic acid fermentation is required for NLRP3 inflammasome activation. Front Immunol (2021) 12:630380. doi: 10.3389/fimmu.2021.630380 33854503 PMC8039150

[68] Colak D Alaiya AA Kaya N Muiya NP AlHarazi O Shinwari Z. Integrated left ventricular global transcriptome and proteome profiling in human end-stage dilated cardiomyopathy. PloS One (2016) 11(10):e0162669. doi: 10.1371/journal.pone.0162669 27711126 PMC5053516

[69] Zhu Y Wu G Yan W Zhan H Sun P. miR-146b-5p regulates cell growth, invasion, and metabolism by targeting PDHB in colorectal cancer. Am J Cancer Res (2017) 7(5):1136–50. PMC5446479 28560062

[70] Sun XR Sun Z Zhu Z Guan HX Li CY Zhang JY. Expression of pyruvate dehydrogenase is an independent prognostic marker in gastric cancer. World J Gastroenterol (2015) 21(17):5336–44. doi: 10.3748/wjg.v21.i17.5336 PMC4419075 25954108

[71] Zhang Z Pang T Qi M Sun G. The biological processes of ferroptosis involved in pathogenesis of COVID-19 and core ferroptoic genes related with the occurrence and severity of this disease. Evol Bioinform Online (2023) 19:11769343231153293. doi: 10.1177/11769343231153293 36820229 PMC9929189

[72] Lu L Liu LP Gui R Dong H Su YR Zhou XH. Discovering common pathogenetic processes between COVID-19 and sepsis by bioinformatics and system biology approach. Front Immunol (2022) 13:975848. doi: 10.3389/fimmu.2022.975848 36119022 PMC9471316

[73] Nguyen Thanh D Thanh Giang NT Le TV Truong NM Ngo TV Lam TN. Predicting the severity of COVID-19 patients using the CD24-CSF1R index in whole blood samples. Heliyon (2023) 9(3):e13945. doi: 10.1016/j.heliyon.2023.e13945 36851954 PMC9946875

[74] Smilowitz NR Kunichoff D Garshick M Shah B Pillinger M Hochman JS. C-reactive protein and clinical outcomes in patients with COVID-19. Eur Heart J (2021) 42(23):2270–9. doi: 10.1093/eurheartj/ehaa1103 PMC7928982 33448289

[75] Hadid T Kafri Z Al-Katib A. Coagulation and anticoagulation in COVID-19. Blood Rev (2021) 47:100761. doi: 10.1016/j.blre.2020.100761 33067035 PMC7543932

