# Supplementary material for: Identification of cuproptosis-related molecular subtypes and a novel predictive model of COVID-19 based on machine learning
Source: Front Immunol. 2023 Jul 17;14:1152223. doi: 10.3389/fimmu.2023.1152223 (PMC10393044; doi:10.3389/fimmu.2023.1152223)

Figure S1 Differences in clinical parameters between the two cuproptosis clusters. (A) Ventilator-free days. (B) The number of patients undergoing mechanical ventilation. (C) The number of patients admitted to the ICU.

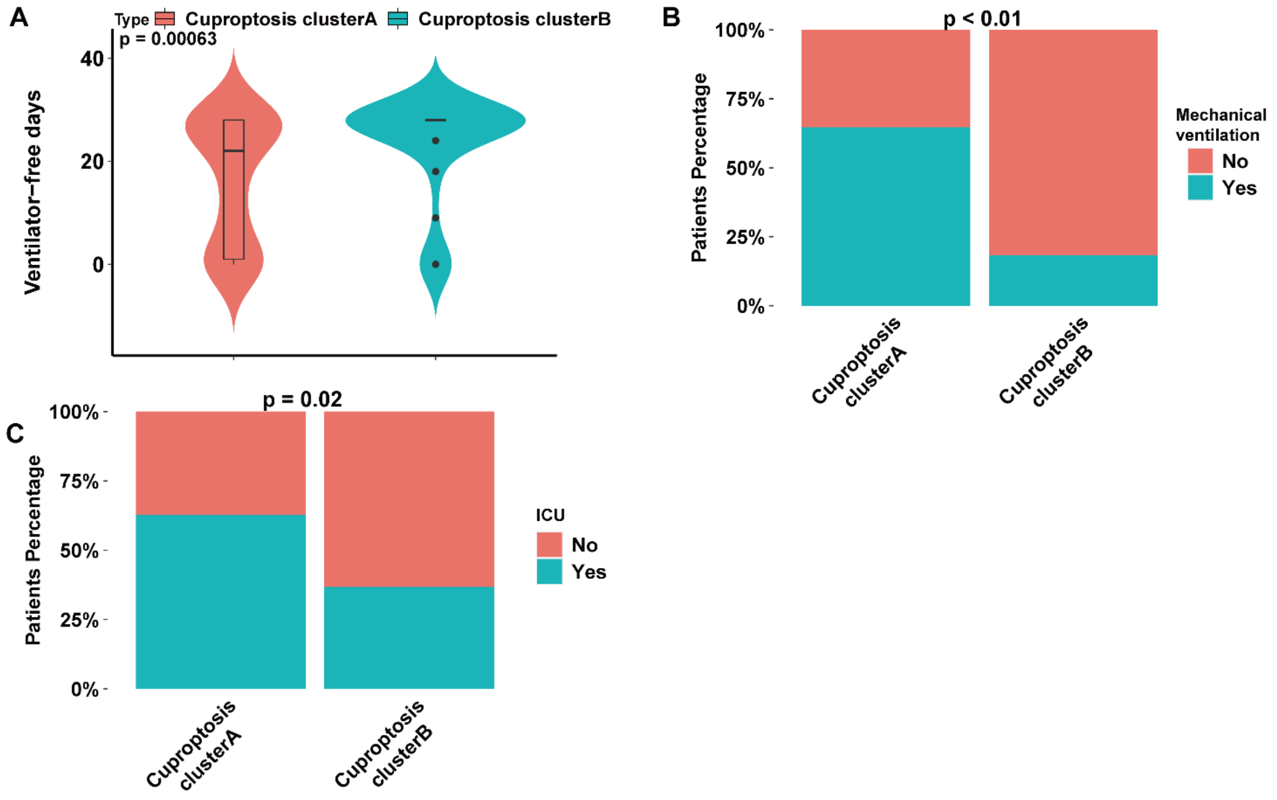

Supplement: Supplementary file 1 [file Image_1.pdf]
